# *N*′-[(*E*)-1-(4-Bromo­phen­yl)ethyl­idene]-4-hy­droxy-2-methyl-1,1-dioxo-2*H*-1,2-benzothia­zine-3-carbohydrazide

**DOI:** 10.1107/S1600536811035641

**Published:** 2011-09-14

**Authors:** Naveed Ahmad, Muhammad Zia-ur-Rehman, Hamid Latif Siddiqui, Muhammad Nadeem Arshad, Abdullah M. Asiri

**Affiliations:** a Institute of Chemistry, University of the Punjab, Lahore 54590, Pakistan; b Applied Chemistry Research Centre, PCSIR Laboratories Complex, Lahore 54600, Pakistan; c X-ray Diffraction and Physical Laboratory, Department of Physics, School of Physical Sciences, University of the Punjab, Lahore 54590, Pakistan; d The Center of Excellence for Advanced Materials Research, King Abdulaziz University, Jeddah, PO Box 80203, Saudi Arabia

## Abstract

The six-membered heterocycle in the title compound, C_18_H_16_BrN_3_O_4_S, adopts a sofa conformation. Intra­molecular N—H⋯N and O—H⋯O hydrogen bonds stabilize the mol­ecular conformation by forming a five- and a six-membered ring, respectively. The crystal packing is stabilized by inter­molecular C—H⋯O hydrogen bonds.

## Related literature

For general background, see: Zia-ur-Rehman *et al.* (2009 ▶). For synthesis details, see: Ahmad *et al.* (2011 ▶). For graph-set notation of hydrogen-bond motifs, see: Bernstein *et al.* (1995 ▶).
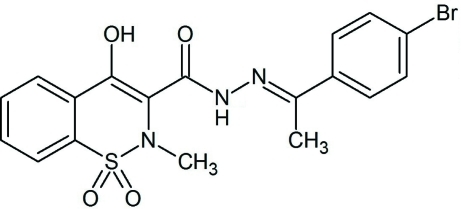


## Experimental

### 

#### Crystal data

C_18_H_16_BrN_3_O_4_S
*M* *_r_* = 450.31
Monoclinic, 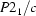

*a* = 14.692 (2) Å
*b* = 16.562 (2) Å
*c* = 7.5254 (10) Å
β = 104.820 (1)°
*V* = 1770.2 (4) Å^3^
*Z* = 4
Mo *K*α radiation
μ = 2.47 mm^−1^
*T* = 173 K
0.48 × 0.36 × 0.11 mm

#### Data collection

Siemens SMART diffractometer equipped with a Bruker KappaCCD APEXII
Absorption correction: multi-scan (*SADABS*; Bruker, 2001 ▶) *T*_min_ = 0.383, *T*_max_ = 0.773
21408 measured reflections
4490 independent reflections
3600 reflections with *I* > 2σ(*I*)
*R*_int_ = 0.034

#### Refinement

*R*[*F* ^2^ > 2σ(*F* ^2^)] = 0.029
*wR*(*F* ^2^) = 0.070
*S* = 1.03
4490 reflections
292 parameters
H atoms treated by a mixture of independent and constrained refinement
Δρ_max_ = 0.44 e Å^−3^
Δρ_min_ = −0.40 e Å^−3^

### 

Data collection: *SMART* (Bruker, 2001 ▶); cell refinement: *SAINT* (Bruker, 2001 ▶); data reduction: *SAINT*; program(s) used to solve structure: *SHELXS97* (Sheldrick, 2008 ▶); program(s) used to refine structure: *SHELXL97* (Sheldrick, 2008 ▶); molecular graphics: *PLATON* (Spek, 2009 ▶); software used to prepare material for publication: *WinGX* (Farrugia, 1999 ▶) and *X-SEED* (Barbour, 2001 ▶).

## Supplementary Material

Crystal structure: contains datablock(s) I, global. DOI: 10.1107/S1600536811035641/bt5633sup1.cif

Structure factors: contains datablock(s) I. DOI: 10.1107/S1600536811035641/bt5633Isup2.hkl

Supplementary material file. DOI: 10.1107/S1600536811035641/bt5633Isup3.cml

Additional supplementary materials:  crystallographic information; 3D view; checkCIF report

## Figures and Tables

**Table 1:** Hydrogen-bond geometry (Å, °)

*D*—H⋯*A*	*D*—H	H⋯*A*	*D*⋯*A*	*D*—H⋯*A*
C17—H17*C*⋯O2^i^	0.95 (3)	2.38 (3)	3.275 (2)	158 (2)
C17—H17*A*⋯O4^ii^	0.95 (3)	2.54 (3)	3.479 (2)	171 (2)
N2—H2*N*⋯N1	0.84 (3)	2.24 (3)	2.690 (2)	114 (2)
O1—H1*O*⋯O4	0.82 (3)	1.86 (3)	2.5979 (18)	148 (3)

Symmetry codes: (i) 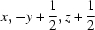; (ii) 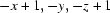.

**Fig. 1.**
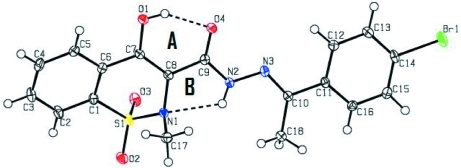
The title molecule with the displacement ellipsoids plotted at 50% probability level (Farrugia, 1999).

**Fig. 2.**
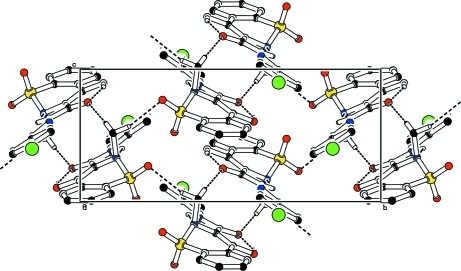
The unit cell packing of the title compound; H bonds have been plotted with dashed lines and H-atoms not involved in hydrogen bonds have been excluded for clarity.

## supplementary crystallographic information

### Comment

In continuation of our on-going research on various biologically active benzothiazine derivatives (Zia-ur-Rehman *et al.*, 2009; Ahmad *et al.*, 2011) synthesis and crystal structure of the title molecule (I) is reported here.

In the crystal structure of the title compound (I), two fused rings (benzene & thiazine) are twisted with a dihedral angle of 13.61 (10)° while the later (C1/C6/C7/C8/N1/S1) adopts half chair conformation [Nitrogen (0.3564 (10)Å and sulfur (-0.3114 (9) Å) atoms show maximum deviation from the least square plane]. The bromophenyl ring (C11—C16) is oriented almost at the same dihedral angle that measures 27.93 (7)° and 26.23 (8)° with respect to the thiazine and aromatic ring (C1—C6). Intramolecular hydrogen bonding through O—H···O and N—H···N interactions gives rise to two different rings *S*_1_^1^(6) A and *S*_1_^1^(5) B respectively (Figure 1). Rings generated from intramolecular hydrogen bondings are fused and twisted at dihedral angle of 5.01 (82)Å and both are inclined at 22.00 (47)Å and 18.83 (27)Å with respect to the thiazine ring. Molecules of the title compound (I) are involved in symmetry related C—H···O weak interactions which form inversion dimers and give rise to the formation of a twelve membered ring *R*_2_^2^(12) (Bernstein *et al.*, 1995). The dimers are further linked through another C—H···O interaction generating from *N*-methyl hydrogen and sulfone oxygen atoms giving rise to two dimensional polymeric network along *bc* plane (Figure 2., Table 1).

### Experimental

A mixture of 4-hydroxy-2*H*-1,2-benzothiazine-3-carbohydrazide 1,1-dioxide (2.0 mmol), 4-bromo acetophenone (2.0 mmol), *ortho* phosphoric acid (2 drops) and methanol (50 ml) was refluxed for a period of seven hours. The content  was cooled to 5°C in an ice bath, filtered and the solids were washed with cold methanol to get the pure compound. The product was crystallized from ethanol to get the suitable crystals. Yield: 82%.

### Refinement

The coordinates of all H atoms  were refined with U(H) set to 1.2*U*_eq_ for all N and aromatic C  atoms and 1.5*U*_eq_ for O and C_methyl_.

### Crystal data

**Table:**

C_18_H_16_BrN_3_O_4_S	*F*(000) = 912
*M**_r_* = 450.31	*D*_x_ = 1.690 Mg m^−^^3^
Monoclinic, *P*2_1_/*c*	Mo *K*α radiation, λ = 0.71073 Å
Hall symbol: -P 2ybc	Cell parameters from 6699 reflections
*a* = 14.692 (2) Å	θ = 2.9–28.6°
*b* = 16.562 (2) Å	µ = 2.47 mm^−^^1^
*c* = 7.5254 (10) Å	*T* = 173 K
β = 104.820 (1)°	Block, light yellow
*V* = 1770.2 (4) Å^3^	0.48 × 0.36 × 0.11 mm
*Z* = 4	

### Data collection

**Table:**

Siemens SMART diffractometer equipped with a Bruker KappaCCD APEXII	4490 independent reflections
Radiation source: fine-focus sealed tube	3600 reflections with *I* > 2σ(*I*)
graphite	*R*_int_ = 0.034
φ and ω scans	θ_max_ = 28.9°, θ_min_ = 1.9°
Absorption correction: multi-scan (*SADABS*; Bruker, 2001)	*h* = −19→19
*T*_min_ = 0.383, *T*_max_ = 0.773	*k* = −22→22
21408 measured reflections	*l* = −10→10

### Refinement

**Table:**

Refinement on *F*^2^	Primary atom site location: structure-invariant direct methods
Least-squares matrix: full	Secondary atom site location: difference Fourier map
*R*[*F*^2^ > 2σ(*F*^2^)] = 0.029	Hydrogen site location: inferred from neighbouring sites
*wR*(*F*^2^) = 0.070	H atoms treated by a mixture of independent and constrained refinement
*S* = 1.03	*w* = 1/[σ^2^(*F*_o_^2^) + (0.0314*P*)^2^ + 1.043*P*] where *P* = (*F*_o_^2^ + 2*F*_c_^2^)/3
4490 reflections	(Δ/σ)_max_ = 0.001
292 parameters	Δρ_max_ = 0.44 e Å^−^^3^
0 restraints	Δρ_min_ = −0.40 e Å^−^^3^

### Special details

**Table:**

Geometry. All s.u.'s (except the s.u. in the dihedral angle between two l.s. planes) are estimated using the full covariance matrix. The cell s.u.'s are taken into account individually in the estimation of s.u.'s in distances, angles and torsion angles; correlations between s.u.'s in cell parameters are only used when they are defined by crystal symmetry. An approximate (isotropic) treatment of cell s.u.'s is used for estimating s.u.'s involving l.s. planes.
Refinement. Refinement of *F*^2^ against ALL reflections. The weighted *R*-factor *wR* and goodness of fit *S* are based on *F*^2^, conventional *R*-factors *R* are based on *F*, with *F* set to zero for negative *F*^2^. The threshold expression of *F*^2^ > 2σ(*F*^2^) is used only for calculating *R*-factors(gt) *etc*. and is not relevant to the choice of reflections for refinement. *R*-factors based on *F*^2^ are statistically about twice as large as those based on *F*, and *R*- factors based on ALL data will be even larger.

### Fractional atomic coordinates and isotropic or equivalent isotropic displacement parameters (Å^2^)

**Table:**

	*x*	*y*	*z*	*U*_iso_*/*U*_eq_	
C1	0.74833 (13)	0.08528 (11)	0.1120 (2)	0.0143 (4)	
C2	0.83074 (14)	0.10149 (12)	0.0597 (3)	0.0192 (4)	
C3	0.87895 (14)	0.03747 (13)	0.0071 (3)	0.0214 (4)	
C4	0.84550 (14)	−0.04104 (12)	0.0080 (3)	0.0191 (4)	
C5	0.76259 (13)	−0.05643 (11)	0.0574 (3)	0.0153 (4)	
C6	0.71272 (12)	0.00689 (11)	0.1115 (2)	0.0125 (3)	
C7	0.62439 (12)	−0.00730 (10)	0.1649 (2)	0.0118 (3)	
C8	0.58824 (12)	0.04814 (10)	0.2620 (2)	0.0126 (3)	
C9	0.49476 (12)	0.03746 (10)	0.2936 (2)	0.0122 (3)	
C10	0.35797 (12)	0.15959 (11)	0.5072 (2)	0.0126 (3)	
C11	0.25982 (12)	0.16068 (11)	0.5279 (2)	0.0127 (3)	
C12	0.19684 (13)	0.09850 (11)	0.4527 (2)	0.0143 (4)	
C13	0.10540 (13)	0.09808 (12)	0.4725 (3)	0.0171 (4)	
C14	0.07621 (12)	0.16057 (12)	0.5683 (3)	0.0168 (4)	
C15	0.13608 (13)	0.22345 (12)	0.6414 (3)	0.0166 (4)	
C16	0.22758 (13)	0.22328 (11)	0.6209 (2)	0.0146 (4)	
C17	0.70484 (14)	0.11010 (12)	0.5184 (3)	0.0177 (4)	
C18	0.42638 (14)	0.22228 (12)	0.6057 (3)	0.0179 (4)	
N1	0.63983 (10)	0.12026 (9)	0.3325 (2)	0.0127 (3)	
N2	0.46422 (11)	0.10249 (9)	0.3728 (2)	0.0132 (3)	
N3	0.37590 (10)	0.10297 (9)	0.4031 (2)	0.0134 (3)	
O1	0.58057 (9)	−0.07719 (8)	0.10541 (17)	0.0144 (3)	
O2	0.74855 (9)	0.22587 (8)	0.26881 (19)	0.0195 (3)	
O3	0.60735 (9)	0.18640 (8)	0.02540 (19)	0.0185 (3)	
O4	0.44888 (9)	−0.02546 (7)	0.25045 (17)	0.0147 (3)	
S1	0.68413 (3)	0.16496 (3)	0.17764 (6)	0.01416 (10)	
Br1	−0.047586 (14)	0.160120 (14)	0.60056 (3)	0.02823 (7)	
H1O	0.530 (2)	−0.0778 (16)	0.134 (4)	0.042*	
H2	0.8547 (18)	0.1523 (15)	0.064 (3)	0.034*	
H2N	0.5020 (19)	0.1414 (16)	0.401 (3)	0.034*	
H3	0.9330 (18)	0.0479 (15)	−0.028 (3)	0.034*	
H4	0.8796 (18)	−0.0832 (15)	−0.022 (3)	0.034*	
H5	0.7398 (17)	−0.1094 (15)	0.056 (3)	0.034*	
H12	0.2183 (17)	0.0564 (15)	0.385 (3)	0.034*	
H13	0.0610 (17)	0.0546 (16)	0.420 (3)	0.034*	
H15	0.1165 (17)	0.2691 (15)	0.707 (3)	0.034*	
H16	0.2668 (18)	0.2657 (15)	0.667 (3)	0.034*	
H17A	0.6686 (19)	0.0859 (16)	0.592 (4)	0.042*	
H17B	0.758 (2)	0.0739 (16)	0.512 (4)	0.042*	
H17C	0.727 (2)	0.1614 (16)	0.566 (4)	0.042*	
H18A	0.488 (2)	0.2076 (17)	0.624 (4)	0.042*	
H18B	0.4183 (18)	0.2313 (16)	0.732 (4)	0.042*	
H18C	0.4163 (19)	0.2714 (17)	0.549 (4)	0.042*	

### Atomic displacement parameters (Å^2^)

**Table:**

	*U*^11^	*U*^22^	*U*^33^	*U*^12^	*U*^13^	*U*^23^
C1	0.0125 (8)	0.0151 (9)	0.0161 (9)	0.0036 (7)	0.0051 (7)	0.0024 (7)
C2	0.0163 (9)	0.0179 (10)	0.0260 (10)	−0.0013 (7)	0.0100 (8)	0.0032 (8)
C3	0.0145 (9)	0.0274 (11)	0.0252 (11)	0.0027 (8)	0.0101 (8)	0.0029 (8)
C4	0.0178 (10)	0.0220 (10)	0.0188 (10)	0.0065 (8)	0.0072 (8)	−0.0013 (8)
C5	0.0173 (9)	0.0162 (9)	0.0125 (9)	0.0020 (7)	0.0039 (7)	−0.0005 (7)
C6	0.0124 (8)	0.0136 (8)	0.0109 (8)	0.0026 (7)	0.0021 (7)	0.0014 (6)
C7	0.0115 (8)	0.0113 (8)	0.0120 (8)	−0.0003 (6)	0.0021 (7)	0.0026 (6)
C8	0.0119 (8)	0.0125 (8)	0.0135 (9)	−0.0008 (7)	0.0032 (7)	0.0011 (7)
C9	0.0131 (8)	0.0140 (8)	0.0093 (8)	0.0013 (7)	0.0025 (6)	0.0032 (6)
C10	0.0120 (8)	0.0143 (8)	0.0110 (8)	−0.0003 (7)	0.0023 (6)	0.0025 (7)
C11	0.0115 (8)	0.0159 (9)	0.0111 (8)	−0.0001 (7)	0.0034 (6)	0.0034 (7)
C12	0.0151 (9)	0.0131 (9)	0.0148 (9)	0.0004 (7)	0.0042 (7)	0.0012 (7)
C13	0.0138 (9)	0.0170 (9)	0.0200 (10)	−0.0018 (7)	0.0035 (7)	0.0005 (7)
C14	0.0099 (8)	0.0218 (9)	0.0196 (9)	0.0012 (7)	0.0055 (7)	0.0039 (7)
C15	0.0168 (9)	0.0188 (9)	0.0152 (9)	0.0020 (7)	0.0057 (7)	−0.0006 (7)
C16	0.0162 (9)	0.0148 (9)	0.0132 (9)	−0.0004 (7)	0.0045 (7)	−0.0004 (7)
C17	0.0179 (10)	0.0177 (10)	0.0171 (10)	−0.0017 (8)	0.0036 (8)	−0.0016 (7)
C18	0.0153 (9)	0.0185 (10)	0.0211 (10)	−0.0032 (7)	0.0067 (8)	−0.0036 (8)
N1	0.0127 (7)	0.0103 (7)	0.0166 (8)	−0.0011 (6)	0.0064 (6)	0.0000 (6)
N2	0.0103 (7)	0.0151 (8)	0.0150 (8)	−0.0007 (6)	0.0045 (6)	−0.0001 (6)
N3	0.0114 (7)	0.0164 (8)	0.0132 (7)	0.0008 (6)	0.0042 (6)	0.0024 (6)
O1	0.0136 (6)	0.0134 (6)	0.0163 (7)	−0.0016 (5)	0.0043 (5)	−0.0008 (5)
O2	0.0179 (7)	0.0126 (6)	0.0297 (8)	−0.0017 (5)	0.0093 (6)	0.0000 (5)
O3	0.0158 (7)	0.0167 (7)	0.0239 (7)	0.0040 (5)	0.0069 (6)	0.0058 (5)
O4	0.0141 (6)	0.0146 (6)	0.0160 (6)	−0.0021 (5)	0.0048 (5)	0.0004 (5)
S1	0.0124 (2)	0.0112 (2)	0.0204 (2)	0.00122 (16)	0.00694 (17)	0.00262 (17)
Br1	0.01334 (10)	0.03465 (13)	0.03960 (14)	0.00017 (8)	0.01204 (9)	−0.00172 (10)

### Geometric parameters (Å, °)

**Table:**

C1—C2	1.392 (3)	C12—C13	1.389 (3)
C1—C6	1.399 (3)	C12—H12	0.96 (3)
C1—S1	1.7646 (18)	C13—C14	1.390 (3)
C2—C3	1.388 (3)	C13—H13	0.98 (3)
C2—H2	0.91 (2)	C14—C15	1.383 (3)
C3—C4	1.391 (3)	C14—Br1	1.8956 (18)
C3—H3	0.91 (3)	C15—C16	1.392 (3)
C4—C5	1.385 (3)	C15—H15	0.99 (3)
C4—H4	0.92 (3)	C16—H16	0.92 (3)
C5—C6	1.398 (2)	C17—N1	1.488 (2)
C5—H5	0.94 (3)	C17—H17A	0.95 (3)
C6—C7	1.472 (2)	C17—H17B	1.00 (3)
C7—O1	1.344 (2)	C17—H17C	0.95 (3)
C7—C8	1.363 (2)	C18—H18A	0.92 (3)
C8—N1	1.441 (2)	C18—H18B	1.00 (3)
C8—C9	1.463 (2)	C18—H18C	0.91 (3)
C9—O4	1.239 (2)	N1—S1	1.6488 (15)
C9—N2	1.361 (2)	N2—N3	1.374 (2)
C10—N3	1.291 (2)	N2—H2N	0.84 (3)
C10—C11	1.490 (2)	O1—H1O	0.82 (3)
C10—C18	1.502 (3)	O2—S1	1.4326 (14)
C11—C16	1.400 (3)	O3—S1	1.4318 (14)
C11—C12	1.403 (2)		
			
C2—C1—C6	122.01 (17)	C12—C13—H13	121.4 (14)
C2—C1—S1	120.07 (14)	C14—C13—H13	119.5 (14)
C6—C1—S1	117.92 (13)	C15—C14—C13	121.17 (17)
C3—C2—C1	118.53 (18)	C15—C14—Br1	119.05 (14)
C3—C2—H2	119.8 (16)	C13—C14—Br1	119.79 (14)
C1—C2—H2	121.6 (16)	C14—C15—C16	119.24 (17)
C2—C3—C4	120.41 (18)	C14—C15—H15	122.6 (14)
C2—C3—H3	118.7 (16)	C16—C15—H15	118.2 (14)
C4—C3—H3	120.8 (16)	C15—C16—C11	121.13 (17)
C5—C4—C3	120.63 (18)	C15—C16—H16	119.1 (16)
C5—C4—H4	119.9 (15)	C11—C16—H16	119.7 (16)
C3—C4—H4	119.5 (15)	N1—C17—H17A	106.1 (16)
C4—C5—C6	120.16 (18)	N1—C17—H17B	110.2 (15)
C4—C5—H5	120.3 (15)	H17A—C17—H17B	110 (2)
C6—C5—H5	119.5 (15)	N1—C17—H17C	109.3 (16)
C5—C6—C1	118.24 (16)	H17A—C17—H17C	110 (2)
C5—C6—C7	121.59 (16)	H17B—C17—H17C	111 (2)
C1—C6—C7	120.17 (15)	C10—C18—H18A	113.8 (17)
O1—C7—C8	122.64 (16)	C10—C18—H18B	110.2 (15)
O1—C7—C6	115.28 (15)	H18A—C18—H18B	105 (2)
C8—C7—C6	122.03 (16)	C10—C18—H18C	112.1 (17)
C7—C8—N1	121.02 (15)	H18A—C18—H18C	110 (2)
C7—C8—C9	121.10 (16)	H18B—C18—H18C	106 (2)
N1—C8—C9	117.86 (15)	C8—N1—C17	113.77 (14)
O4—C9—N2	124.22 (16)	C8—N1—S1	112.18 (12)
O4—C9—C8	121.97 (16)	C17—N1—S1	116.15 (12)
N2—C9—C8	113.81 (15)	C9—N2—N3	120.56 (15)
N3—C10—C11	115.16 (16)	C9—N2—H2N	116.5 (18)
N3—C10—C18	125.93 (16)	N3—N2—H2N	123.0 (18)
C11—C10—C18	118.91 (16)	C10—N3—N2	116.92 (15)
C16—C11—C12	118.21 (16)	C7—O1—H1O	108.1 (19)
C16—C11—C10	121.45 (16)	O3—S1—O2	119.83 (8)
C12—C11—C10	120.34 (16)	O3—S1—N1	107.72 (8)
C13—C12—C11	121.10 (17)	O2—S1—N1	108.04 (8)
C13—C12—H12	120.6 (15)	O3—S1—C1	109.24 (9)
C11—C12—H12	118.3 (15)	O2—S1—C1	109.01 (8)
C12—C13—C14	119.14 (17)	N1—S1—C1	101.43 (8)
			
C6—C1—C2—C3	−0.5 (3)	C11—C12—C13—C14	0.1 (3)
S1—C1—C2—C3	−179.37 (15)	C12—C13—C14—C15	1.0 (3)
C1—C2—C3—C4	−0.4 (3)	C12—C13—C14—Br1	−178.89 (14)
C2—C3—C4—C5	1.4 (3)	C13—C14—C15—C16	−1.0 (3)
C3—C4—C5—C6	−1.6 (3)	Br1—C14—C15—C16	178.84 (14)
C4—C5—C6—C1	0.7 (3)	C14—C15—C16—C11	0.0 (3)
C4—C5—C6—C7	179.99 (17)	C12—C11—C16—C15	1.1 (3)
C2—C1—C6—C5	0.3 (3)	C10—C11—C16—C15	−179.30 (17)
S1—C1—C6—C5	179.21 (13)	C7—C8—N1—C17	−88.2 (2)
C2—C1—C6—C7	−178.95 (17)	C9—C8—N1—C17	93.45 (19)
S1—C1—C6—C7	−0.1 (2)	C7—C8—N1—S1	46.2 (2)
C5—C6—C7—O1	−19.8 (2)	C9—C8—N1—S1	−132.15 (14)
C1—C6—C7—O1	159.41 (16)	O4—C9—N2—N3	−2.8 (3)
C5—C6—C7—C8	162.47 (17)	C8—C9—N2—N3	176.94 (15)
C1—C6—C7—C8	−18.3 (3)	C11—C10—N3—N2	176.67 (14)
O1—C7—C8—N1	176.41 (15)	C18—C10—N3—N2	−3.4 (3)
C6—C7—C8—N1	−6.1 (3)	C9—N2—N3—C10	167.98 (16)
O1—C7—C8—C9	−5.3 (3)	C8—N1—S1—O3	60.35 (14)
C6—C7—C8—C9	172.21 (16)	C17—N1—S1—O3	−166.41 (13)
C7—C8—C9—O4	7.4 (3)	C8—N1—S1—O2	−168.87 (12)
N1—C8—C9—O4	−174.25 (15)	C17—N1—S1—O2	−35.63 (15)
C7—C8—C9—N2	−172.33 (16)	C8—N1—S1—C1	−54.33 (13)
N1—C8—C9—N2	6.0 (2)	C17—N1—S1—C1	78.91 (14)
N3—C10—C11—C16	−173.38 (16)	C2—C1—S1—O3	98.82 (17)
C18—C10—C11—C16	6.6 (3)	C6—C1—S1—O3	−80.10 (16)
N3—C10—C11—C12	6.2 (2)	C2—C1—S1—O2	−33.83 (18)
C18—C10—C11—C12	−173.73 (17)	C6—C1—S1—O2	147.26 (14)
C16—C11—C12—C13	−1.1 (3)	C2—C1—S1—N1	−147.64 (16)
C10—C11—C12—C13	179.26 (16)	C6—C1—S1—N1	33.45 (16)

### Hydrogen-bond geometry (Å, °)

**Table:**

*D*—H···*A*	*D*—H	H···*A*	*D*···*A*	*D*—H···*A*
C17—H17C···O2^i^	0.95 (3)	2.38 (3)	3.275 (2)	158 (2)
C17—H17A···O4^ii^	0.95 (3)	2.54 (3)	3.479 (2)	171 (2)
N2—H2N···N1	0.84 (3)	2.24 (3)	2.690 (2)	114 (2)
O1—H1O···O4	0.82 (3)	1.86 (3)	2.5979 (18)	148 (3)

Symmetry codes: (i) *x*, −*y*+1/2, *z*+1/2; (ii) −*x*+1, −*y*, −*z*+1.
